# Sera Metabolomics Characterization of Patients at Different Stages in Wuhan Identifies Critical Biomarkers of COVID-19

**DOI:** 10.3389/fcimb.2022.882661

**Published:** 2022-05-02

**Authors:** Meijia Gu, Huaqin Pan, Yuncong Yuan, Xuemin Zhou, Luojia Chen, Xingran Wang, Fang Fang, Liu Hu, Yaxuan Xie, Chao Shen

**Affiliations:** ^1^ Key Laboratory of Combinatorial Biosynthesis and Drug Discovery, Ministry of Education, School of Pharmaceutical Sciences, Wuhan University, Wuhan, China; ^2^ Department of Critical Care Medicine, Zhongnan Hospital of Wuhan University; Clinical Research Center of Hubei Critical Care Medicine, Wuhan, China; ^3^ College of Life Sciences, Wuhan University, Wuhan, China; ^4^ China Center for Type Culture Collection, Wuhan University, Wuhan, China; ^5^ Shanghai BIOTREE Biological Technology Co., Ltd, Shanghai, China; ^6^ Department of Laboratory Medicine, Zhongnan Hospital of Wuhan University, Wuhan, China; ^7^ School of Health Sciences, Wuhan University, Wuhan, China

**Keywords:** COVID-19, metabolomics, biomarkers, stages, sera

## Abstract

We have witnessed the 2-year-long global rampage of COVID-19 caused by the wide spread of severe acute respiratory syndrome coronavirus 2 (SARS-CoV-2). However, knowledge about biomarkers of the entire COVID-19 process is limited. Identification of the systemic features of COVID-19 will lead to critical biomarkers and therapeutic targets for early intervention and clinical disease course prediction. Here, we performed a comprehensive analysis of clinical measurements and serum metabolomics in 199 patients with different stages of COVID-19. In particular, our study is the first serum metabolomic analysis of critical rehabilitation patients and critical death patients. We found many differential metabolites in the comparison of metabolomic results between ordinary, severe, and critical patients and uninfected patients. Through the metabolomic results of COVID-19 patients in various stages, and critical rehabilitation patients and critical death patients, we identified a series of differential metabolites as biomarkers, a separate queue and precise distinction, and predicted COVID-19 verification. These differentially expressed metabolites, included 1,2-di-(9Z,12Z-octadecadienoyl)-sn-glycero-3-phosphate, propylparaben, 20-hydroxyeicosatetraenoic acid, triethanolamine, chavicol, disialosyl galactosyl globoside, 1-arachidonoylglycerophosphoinositol, and alpha-methylstyrene, all of which have been identified for the first time as biomarkers in COVID-19 progression. These biomarkers are involved in many pathological and physiological pathways of COVID-19, for example, immune responses, platelet degranulation, and metabolism which might result in pathogenesis. Our results showed valuable information about metabolites obviously altered in COVID-19 patients with different stages, which could shed light on the pathogenesis as well as serve as potential therapeutic agents of COVID-19.

## Introduction

The coronavirus disease 2019 (COVID-19), which first broke out in Wuhan, has lasted over 3 years, causing a global health and economic development crisis (Acter et al., 2020; Peeri et al., 2020; Shu et al., 2020). So far, the COVID-19 pandemic has resulted in more than 354,034,021 reported confirmed cases, with 5,602,814 global deaths (data were from Johns Hopkins University up to 25 January 2022). Thus, different types of drugs, vaccines, and treatments against COVID-19 are also being developed in many recent studies (Gu et al., 2021; Huang et al., 2021; Wu et al., 2021).

COVID-19 is an acute respiratory infectious disease due to highly infectious severe acute respiratory syndrome coronavirus 2 (SARS-CoV-2) (Bchetnia et al., 2020; Huang et al., 2020; Park, 2020). SARS-CoV-2 is a coronavirus belonging to the β genus (Jangra and Saxena, 2020; Yeo et al., 2020). It is enveloped and has round or elliptical particles with diameters of 60 ~ 140 nm (Nikhra, 2020; Laue et al., 2021). It has five essential genes for four structural proteins, namely spiking protein (S), viral envelope (E), nucleoprotein (N), matrix protein (M), and RNA-dependent RNA polymerase (RDRP) (Naqvi et al., 2020; Shamsi et al., 2021; Zeng et al., 2021). Through binding to angiotensin-converting enzyme 2 (ACE-2), the spiking protein enters cells. SARS-CoV-2 principally attacks the lower respiratory tract and lung tissue (Chung et al., 2020; Fu et al., 2020; Parsamanesh et al., 2020). Subsequent studies have found that it partly influences all systems of the body. After virus infection, the main manifestations are non-specific symptoms, for instance, fever, dry cough, and fatigue, some patients even have rhinobyon, running rhinorrhea, sore throat, conjunctivitis, myalgia, and diarrhea (Chen N. et al., 2020; Huang et al., 2020; Tang et al., 2021). According to clinical manifestations and signs, patients can be classified into mild disease, severe disease, and critical disease. However, it is often too late to determine which stages patients are in (Desmet et al., 1994; Vilstrup et al., 2014; Shen et al., 2020). In particular, the fifth-generation variant of the new SARS-CoV-2, the Omicron strain, was first detected in South Africa on 9 November 2021. As of January 2022, the Omicron strain has spread to over 128 territories and countries. Compared to the Delta strain, the Omicron strain has had more confirmed infectious, with an R-value of 4.2, which means that each patient of the Omicron strain could infect 4.2 people (Wami et al., 2021). Nevertheless, the Delta strain has an infectivity R-value of only between 1.1 and 1.2 (Del Rio et al., 2021). The high infectivity of the Omicron strain is most likely due to the fact that the Omicron strain infects and duplicates 70 times faster in the bronchi than the Delta strain and the original SARS-CoV-2 strain (Dejnirattisai et al., 2022; Zhang et al., 2022). However, it is promising to note that lung infection caused by the Omicron strain is significantly less dangerous than that of the Delta strain as well as the original SARS-CoV-2 strain, which is a key indicator of the severity of the disease, and this explains to some extent the predominance of patients infected with the Omicron strain who are just ordinary patients (Chen et al., 2022; Hu et al., 2022; Narowski et al., 2022). Although great efforts have been made to investigate human coronavirus disease, the molecular mechanisms of the pathogenesis of COVID-19 are still poorly studied (Pennisi et al., 2020; Ritter et al., 2020; Yang et al., 2020). Further, the clinical features and biomarkers between severely affected and not severely affected cases have still not yet been well clarified (Bauer et al., 2006; Varadkar et al., 2014). Therefore, new methods need to be explored to prevent the deterioration of COVID-19.

Following genomics and proteomics research ideas, a metabolomics study is a quantitative analysis method carried out on all metabolites in the organism that finds the metabolites’ relative relationship with the physiological and pathological changes (Raamsdonk et al., 2001; Chen Y. et al., 2020; Thomas et al., 2020). Because metabolic profiles are unique to each individual, fluctuations and differences in metabolite levels could reflect the body state and the underlying mechanisms of the disease directly (Holmes et al., 2008; Jenab et al., 2009; Wilkins and Trushina, 2017). Several studies have been proposed to explain the diagnosis and prognostic profiling of metabolomics in selecting patient populations (Fraser et al., 2020; Lauc and Sinclair, 2020). Some scholars also studied the lipid metabolism under COVID-19 infection (Abu-farha et al., 2020; Di et al., 2020). In addition, metabolomics was performed to further explore some markers that could indicate the progression and prognosis of COVID-19 (Blasco et al., 2020; Bruzzone et al., 2020; Ponti et al., 2020). Undoubtedly, there is an urgent need to uncover the biomarkers of COVID-19 patients that will enhance the capabilities of facing life-threatening illness so that medical resources can be optimally allocated and rapid treatment can be administered early in the disease course (Weinkove et al., 2020; Weiss et al., 2020; Sindelar et al., 2021). From another aspect, metabolomics phenotyping might represent an important step toward personalized therapeutics in patients infected with COVID-19, because it could predict the antiviral drug efficacy (Migaud et al., 2020a; Migaud et al., 2020b; Gómez-Cebrián et al., 2021). However, previous studies on the metabolomics characterization of COVID-19 mostly focused on the differences of infected populations (Zhao et al., 2020; Lou et al., 2021; Wang et al., 2021). Thus, the molecular alterations in COVID-19 patients with different stages remain to be investigated, and such an effort will lead to a deep understanding of the detailed mechanisms underlying the different clinical stages of COVID-19

**Figure 1:**
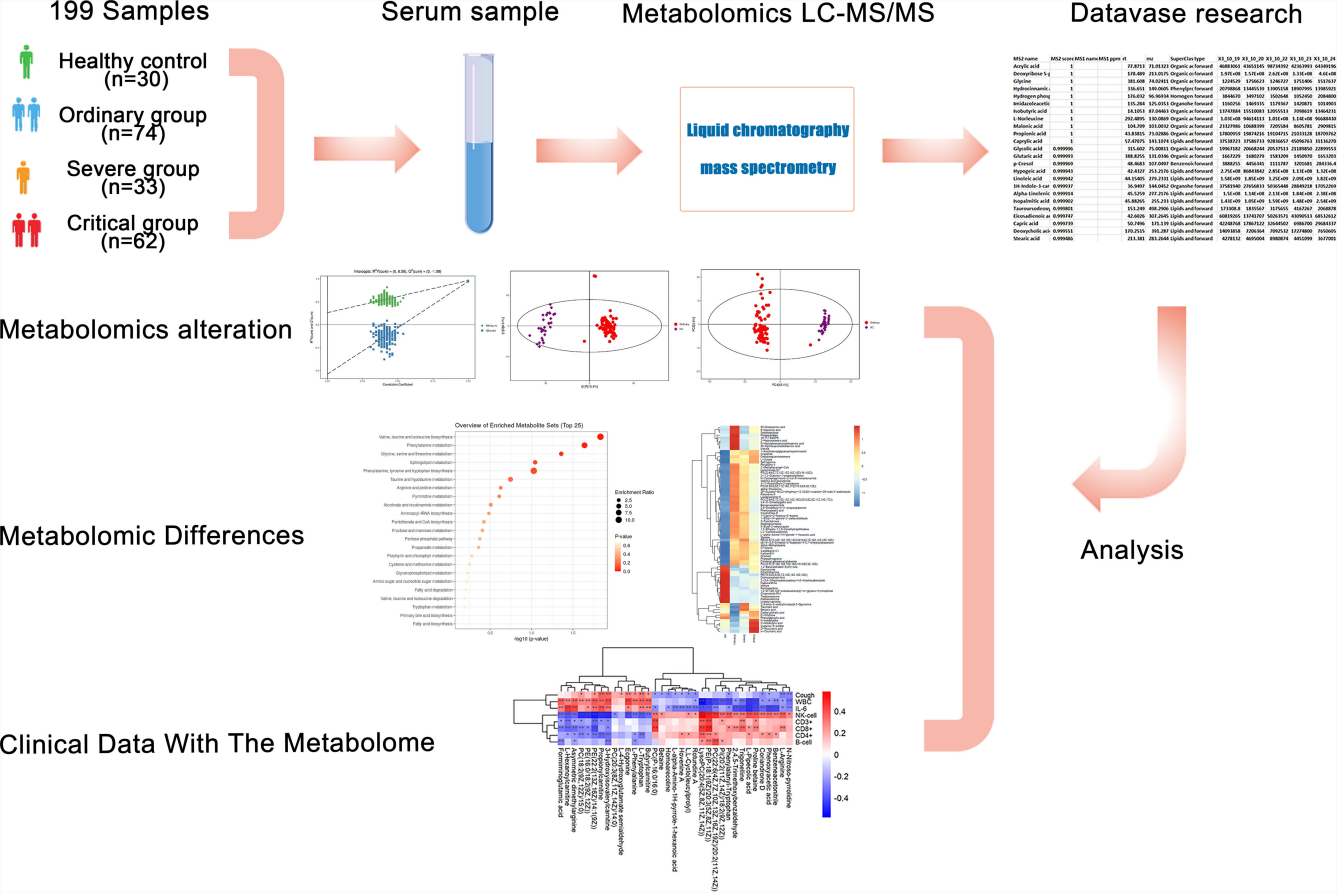
Study design and metabolomics profiling of COVID-19 sera. Overview of cohort (including 30 healthy control samples, 74 ordinary COVID-19 patients, 33 severe COVID-19 patients, and 62 critical COVID-19 patients), and the study design (including metabolomics LC-MS/MS, database search, clinical data, and analysis).

Based upon the severe situation of continuous spread of SARS-CoV-2, we collected fresh serum from 146 COVID-19 patients from Zhongnan Hospital of Wuhan University from 1 January 2020 to 12 March 2020. By performing non-target metabolomics, we figured out the host responses to SARS-CoV-2 infection, with the sera samples from a cohort of COVID-19 patients, including the non-survivors (fatalities) and survivors who recovered from critical or severe symptoms. This study revealed a set of COVID-19-related alterations of host metabolites. According to the patient’s condition, a total of 199 samples were classified into four groups as follows: 74 samples in the ordinary group, 30 samples in the healthy control (HC) group, 33 samples in the severe group, and 62 samples in the critical group (**Figure 1**). We performed non-target metabolomics tests on all samples, screened for differential metabolites among them, and conducted hierarchical cluster analysis, differential metabolic pathway analysis, and ROC analysis. In addition, we also analyzed the clinical index data of all these samples, including fever, cough, WBC, IL-6, CRP, etc. Spearman correlation analysis was performed between clinical indicators and differential metabolites. We characterized the dysregulated metabolic pathways and cytokine/chemokine levels in COVID-19 patients compared with healthy controls. Then we demonstrated the escalated correlations between cytokines/chemokines and circulating metabolites from the ordinary to severe groups, shedding lighter on the metabolic pathways and diagnostic biomarkers related to poor prognosis in the course of COVID-19. We aimed to investigate the possible underlying pathogenesis between different disease courses in patients with new coronary disease through metabolomics. Furthermore, these biomarkers, which play crucial roles in main pathological pathways, and the variations of these metabolomics in patients’ sera, contribute to the pathogenesis of COVID-19. These findings provided worthwhile information about sera biomarkers related to COVID-19 and potential therapeutic targets.

## Materials and Methods

### Patients and Samples

A total of 146 COVID-19 patients, who were laboratory-confirmed and diagnosed with COVID-19 pneumonia according to the WHO interim guidance, from 1 January 2020 to 12 March 2020 at Zhongnan Hospital of Wuhan University, Wuhan, China, were enrolled in this study. This study was approved by the Medical Ethical Committee of Zhongnan Hospital of Wuhan University (NO. 2020020K). Diagnosis of SARS-CoV-2 infection was based on the New Coronavirus Pneumonia Prevention and Control Program (6th edition) published by the National Health Commission of China. Healthy subjects were recruited from healthcare workers at Zhongnan Hospital of Wuhan University.

Patients were confirmed to be infected with SARS-CoV-2 by real-time polymerase chain reaction (RT-PCR) on nasal and pharyngeal swab specimens. The throat swab tests and serological tests of the healthy control group were negative for SARS-CoV-2. All blood samples were collected after fasting overnight and by blood collection tubes without anticoagulant. Briefly, the presence of SARS-CoV-2 in pharyngeal swab specimens was detected by RT-PCR using an ORF1ab/N gene PCR kit (Biogerm, Shanghai, China) as described previously (Wang et al., 2020).

In general, all the blood samples of patients were treated following the biocontainment procedures of the standard requirements of a SARS-CoV-2-positive sample. Briefly, all collected serum samples were subjected to a metal bath at 56°C for 30 min, then kept in 80°C for further analysis. Blood samples (≤3 mL) of COVID-19 patients with severe and mild symptoms were collected when the disease were most serious (3-7 days after hospitalization, before discharge). Blood samples (≤3 mL) of critical COVID-19 patients were collected during the course of their disease at intervals of 3-5 days. Meanwhile, healthy control samples were collected from healthy volunteers recruited from healthcare workers at Zhongnan Hospital of Wuhan University. Each serum sample was treated as described previously (Li et al., 2021). Finally, the supernatant (≥200 μL) of serum was transferred to a new tube for quality control (QC).

For metabolomics data collection, a UHPLC system (Vanquish, Thermo Fisher Scientific) with a UPLC BEH Amide column (2.1 mm × 100 mm, 1.7 μm) coupled to a Q Exactive HFX mass spectrometer (Orbitrap MS, Thermo) was used for further analysis. The mobile phase A was 25 mmol/L of ammonium acetate and 25 mmol/L of ammonia hydroxide in water (pH = 9.75). The mobile phase B was acetonitrile. The elution gradient was set as follows: 0~0.5 min, 95% B; 0.5~7.0 min, 95%~65% B; 7.0~8.0 min, 65%~40% B; 8.0~9.0 min, 40% B; 9.0~9.1 min, 40%~95% B; 9.1~12.0 min, 95% B. The column temperature was 30°C while the auto-sampler temperature was 4 °C. The injection volume of both positive and negative ion modes was 2 μL.

The QE HFX mass spectrometer was used for its ability to acquire MS/MS spectra using the information-dependent acquisition (IDA) mode in the control of the acquisition software (Xcalibur, Thermo). In this mode, the acquisition software continuously evaluates the full-scan MS spectrum. The ESI source conditions were set as follows: sheath gas flow rate as 50 Arb, Aux gas flow rate as 10 Arb, capillary 320°C, and spray voltage as 3.5 kV (positive) or -3.2 kV (negative), respectively.

### Clinical Data Procedures and Collection

We checked chest radiography, clinical charts, laboratory findings, and nursing records for all patients with laboratory-confirmed COVID-19 pneumonia. The data of epidemiological, clinical, laboratory, and radiological features, treatment, and outcomes were obtained from standardized data collection forms and electronic medical records. The severity of COVID-19 was defined based on the international guidelines for community-acquired pneumonia (Metlay et al., 2019; Li et al., 2020; Ye et al., 2020). Two researchers independently reviewed the data to double check the accuracy of collected data. For the data that were not available for records, the researchers directly communicated with patients and doctors to ascertain data integrity. Nucleic acid tests for SARS-CoV-2 were repeated twice and showed virus clearance before the discharge of patients. The clinical outcomes (i.e., discharges, mortality, and hospitalization) were followed up to 30 March 2020.

### Clinical Definitions

Clinical classification was defined according to the COVID-19 Treatment Guidelines (National Health Commission of the People’s Republic of China). A confirmed case of SARS-CoV-2 infection was defined as an individual with throat swabs positive for SARS-CoV-2 nucleic acid by RT-PCR as described above. Severe COVID-19 cases were those meeting any of the following criteria: (1) respiratory distress (≥30 times/min), (2) oxygen saturation ≤  93% at rest, and (3) the arterial partial pressure of oxygen (PaO_2_)/the fraction of inspired oxygen (FiO_2_) ≤ 300 mmHg. Critical COVID-19 patients were defined as the occurrence of respiratory failure requiring mechanical ventilation or shock or other organ failure. Ordinary COVID-19 patients were defined as having fever, respiratory symptoms, etc., with imaging features of pneumonia but could not be classified as severe. Symptom onset date was defined as the date on which symptoms were first observed. Symptoms included fever, fatigue, dry cough, inappetence, myalgia, dyspnea, expectoration, sore throat, diarrhea, nausea, dizziness, headache, abdominal pain, chill, rhinorrhea, chest stuffiness, or nasal congestion.

The exclusion criteria of comorbidities included hypertension, coronary heart disease, diabetes, chronic obstructive pulmonary disease, malignancy, surgical history, chronic kidney disease, cerebrovascular disease, immunodeficiency disease, chronic hepatitis, and tuberculosis. Demographic data and laboratory indicators are shown in **Table S1**. All patients were diagnosed following the guidelines for COVID-19 diagnosis and treatment (Trial Version 7) released by the National Health Commission of the People’s Republic of China based on the course of illness (Arunachalam et al., 2020). The patients were classified into four groups according to disease severity, i.e., critical, severe, ordinary, and healthy control. Critical disease was defined with at least one of the following conditions: (1) ARDS requiring mechanical ventilation, (2) shock, and (3) other organ failure requiring ICU admission. Severe disease was defined with at least one of the following conditions: (1) respiratory rate ≥ 30 times/min, (2) oxygen saturation ≤ 93% at resting state, (3) arterial partial pressure of oxygen (PaO_2_)/fraction of inspired oxygen (FiO_2_) ≤300 mmHg, and (4) pulmonary imaging showing significant progression of lesions by more than 50% within 24-48 h. Ordinary disease was defined as patients with mild clinical symptoms but not reaching the definition of severe disease. Healthy control was defined as patients with normal body temperature and without any respiratory symptoms. The non-anticoagulated venous blood samples were subjected to a metal bath at 56°C for 30 min and then separated by centrifugation at 3000 rpm for 7 min at room temperature after standard diagnostic tests. Whole blood cells were stored at −80°C. A 200-μL aliquot of serum was added to 800 μL of ice-cold methanol, mixed well, and stored at −80°C. Another 200 μL aliquot of serum was added to 800 μL of ice-cold isopropanol, mixed well, and stored at −80°C.

### Raw Data

All raw LC-MS/MS data were deposited to iProX under the accession number: MTBLS3529.

### Statistics

MS raw data files were converted to the mzXML format by ProteoWizard software (version 3.0.19282), and processed by R package XCMS (v3.2) for metabolomics data, respectively. The data pretreatments included peak identification, peak alignment, peak extraction, retention time correction, and peak integration. Then an in-house MS2 database (BiotreeDB) was applied for metabolite annotation. The cutoff for annotation was set at 0.3.

Statistical significance was analyzed using one-tailed Student’s t test or Fisher’s exact test, and P < 0.05 was considered to be statistically significant. The P value was corrected for multiple testing *via* false-discovery rate (FDR) using the Benjamini-Hochberg method.

## Results

### Study Design and Patients

COVID-19 patients with different disease severity have different molecular characteristics. For in-depth understanding, we profiled 199 serum samples (30 healthy control samples, 74 ordinary COVID-19 samples, 33 severe COVID-19 samples, and 62 critical COVID-19 samples) from 30 healthy control, 59 ordinary, 29 severe, and 28 critical COVID-19 patients through targeted and untargeted metabolomics analyses (**Figure 1**). The median years of age were 56.0 (44.5-62.0), 69.0 (57.0-81.0), and 62.0 (52.2-72.5) for ordinary, severe, and critical patients, respectively. The cohort patients’ baseline demographic characteristics, comorbidities, and laboratory values are reported in **Table S1**.

We used the collected sera to perform untargeted metabolomics testing, screened for differential metabolites, and performed hierarchical cluster analysis, differential metabolic pathway analysis, and ROC analysis. At the same time, we collected clinical indicators (such as fever, cough, WBC, IL-6, CRP), performed Spearman correlation analysis on clinical indicators and differential metabolites, and finally studied the diagnostic biomarkers and possible potential pathogenesis of patients with COVID-19 through metabolomics.

Through data preprocessing, we identified 262 metabolites from 5643 metabolite features obtained from the raw data acquired in negative-ionization modes (**Table S2**), while 334 metabolites were identified from 7409 metabolite features extracted from the raw data acquired in positive-ionization modes (**Table S3**). Due to the condition of the disease, all the patients were divided into four groups, healthy control (3), ordinary (74), severe (33), and critical (62). p2 is an MS of the procedure on the machine. MS raw data files were converted from the ProteoWizard software (version 3.0.19282) to the mzXML format and processed by the R package XCMS (v3.2) for metabolic data.

### Metabolomic Alternations Associated With Clinical Symptoms of COVID-19 Compared With Healthy Control

**Figure 2:**
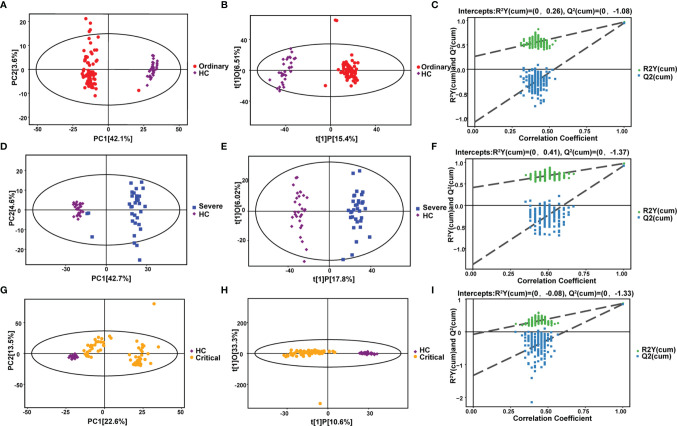
Metabolomic alternations associated with clinical symptoms of COVID-19 compared with healthy control. **(A)** PCA analysis. **(B)** OPLS-DA analysis. **(C)** Permutation analysis. Metabolomic alternations between severe group and healthy control. **(D)** PCA analysis. **(E)** OPLS-DA analysis. **(F)** Permutation analysis. Metabolomic alternations between critical group and healthy control. **(G)** PCA analysis. **(H)** OPLS-DA analysis. **(I)** Permutation analysis.

In order to explain the metabolomic differences between COVID-19 patients and healthy people. Negative ion differential metabolites in the ordinary group, severe group, and critical group were compared with the healthy control group, with the number of differential metabolites shown in each section in **Figure 2**. Altogether, there were 77 common differentially expressed metabolites, including 55 upregulated and 22 downregulated (**Table S4**).

Pattern recognition included pre-processing, extraction and selection of data features, and the building and validation of the data model. We intuitively observed whether the analyzed samples had natural groupings, checking for abnormal samples (points outside the confidence interval), revealing hidden biases present in the study, and showing the detailed information about sample classification.

Our results indicated that the samples were well separated, while the sample point diagram shows a certain degree of aggregation according to the grouping of samples indicating a relatively high-quality confidence. And PLS-DA methods combined with regression models were used to reduce dimensionality, and certain discriminant thresholds were used for discriminant analysis of regression results. From this, we obtained the up and downregulated differential metabolites between the various groups (**Table 1**).

**Table 1:** Metabolomic alternations associated with clinical symptoms of COVID-19 compared with healthy control (HC).

Strategy		Metabolites (P<0.05)	Upregulated	Downregulated	Max (log2FC)	Min (log2FC)
Ordinary VS HC	153		96	57	19	-5.52
Severe VS HC	182		103	79	18.89	-5.8
Critical VS HC	176		102	74	18.23	-4.81

Several differences could be seen from the PCA chart between the ordinary group and HC group (**Figures 2A**–**C**), indicating considerable diversity in metabolism between the ordinary group and healthy control group. OPLS-DA model analysis showed that the two groups were significantly different. The permutation test showed that the OPLS-DA model had no overfitting, confirming the difference between the groups was significant (P<0.05). In NEG mode, we screened 1314 differential peaks, among which there were 81 with the MS2 name, 41 were upregulated, and 40 were downregulated (VIP>1, P<0.05). In POS mode, we selected 2021 differential peaks, among which 72 were MS2, 55 were upregulated, and 17 were downregulated (VIP>1, P<0.05).

It could be distinguished from the PCA chart between the severe group and HC group (**Figures 2D**–**F**) that a significant difference in metabolism occurred between the two groups. OPLS-DA model analysis showed that the two groups were significantly different. The permutation test showed that the OPLS-DA model had no overfitting, confirming the difference between the groups was significant (P<0.05). In NEG mode, we screened 1554 differential peaks, of which 102 were MS2, 48 were upregulated, and 54 were downregulated (VIP>1, P<0.05). In POS mode, we screened 2385 differential peaks, of which 80 were MS2, 55 were upregulated, and 25 were downregulated (VIP>1, P<0.05).

The results were partly disparate in the PCA chart between the critical group and HC group (**Figures 2G**–**I**). Some samples were indistinguishable from HC group samples, indicating that there was a certain difference in metabolism. Some samples had similar metabolic levels to the HC group. OPLS-DA model analysis showed that the two groups were different. The permutation test showed that the OPLS-DA model had no overfitting, confirming the difference between the groups was partly significant (P<0.05). In NEG mode, we screened 566 differential peaks, of which 42 were MS2, 29 were upregulated, and 13 were downregulated (VIP>1, P<0.05). In POS mode, we screened 2961 differential peaks, of which 134 were MS2, 73 were upregulated, and 61 were downregulated (VIP>1, P<0.05).

### Metabolomics Alteration Associated With the Progression of COVID-19

**Figure 3:**
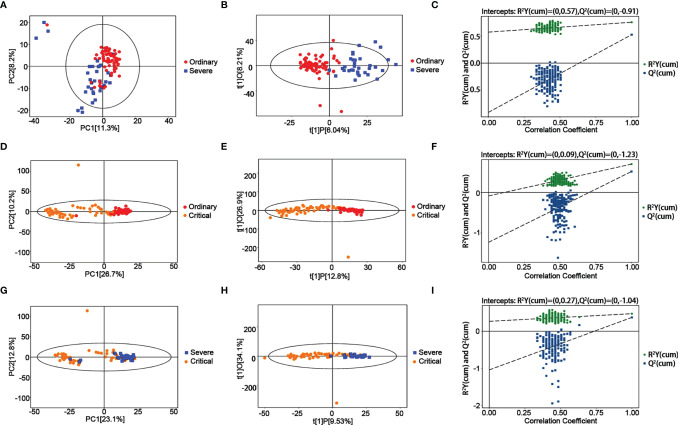
Metabolomic alternations between the severe group and ordinary group as COVID-19 progresses. **(A)** PCA analysis. **(B)** OPLS-DA analysis. **(C)** Permutation analysis. Metabolomic alternations between the critical group and ordinary group. **(D)** PCA analysis. **(E)** OPLS-DA analysis. **(F)** Permutation analysis. Metabolomic alternations between the critical group and severe group. **(G)** PCA analysis. **(H)** OPLS-DA analysis. **(I)** Permutation analysis.

Negative ion differential metabolites in the severe group compared with the critical group are shown in **Figure 3** and **Table 2**, with the number of differential metabolites shown in each section. Altogether, there were 77 common differentially expressed metabolites, including 55 upregulated and 22 downregulated. There were many differences from the PCA chart between the severe group and critical group (**Figure 3**). We could see partial differences from the PCA chart between the severe group and ordinary group (**Figures 3A**–**C**). Some samples were indistinguishable from the ordinary group samples, indicating that there was a certain difference in metabolism. Some samples had similar metabolic levels to the ordinary group. The permutation test showed that the OPLS-DA model had no overfitting, confirming the difference between the groups was significant (P<0.05). In NEG mode, we screened 1043 differential peaks, among which there were 54 with the MS2 name, 27 were upregulated, and 27 were downregulated (VIP>1, P<0.05). In POS mode, we selected 1155 differential peaks, among which 79 were MS2, 22 were upregulated, and 57 were downregulated (VIP>1, P<0.05).

**Table 2:** Metabolomic alternations as COVID-19 process.

Strategy	Metabolites(P<0.05)	Upregulated	Downregulated	Max(log2FC)	Min(log2FC)
Severe VS ordinary	133	49	84	3.43	-2.77
Critical VS ordinary	149	51	98	7.41	-2.06
Critical VS severe	126	63	63	4.39	-1.81

A significant trend of differentiation between the critical and ordinary groups was found from the PCA chart (**Figures 3D**–**F**), and some samples could not be distinguished from the ordinary group, indicating that there was a certain difference in the metabolic level between the severe group and the ordinary group, and some samples were similar to those from the ordinary group. OPLS-DA model analysis showed that the two groups were different. The permutation test showed that the OPLS-DA model had no overfitting, confirming the difference between the groups was partly significant (P<0.05). In NEG mode, we screened 526 differential peaks, of which 28 were MS2, 20 were upregulated, and 8 were downregulated (VIP>1, P<0.05). In POS mode, we screened 3066 differential peaks, of which 121 were MS2, 31 were upregulated, and 90 were downregulated (VIP>1, P<0.05).

Further, there was also a trend of differentiation between the critical and severe groups from the PCA chart (**Figures 3G**–**I**), and some samples could not be distinguished from the severe group, indicating that there was a certain difference in the metabolic levels between the severe group and the moderate group, and some samples were similar to those from the severe group. OPLS-DA model analysis showed that the two groups were significantly different. The permutation test showed that the OPLS-DA model had no overfitting, confirming the difference between the groups was significant (P<0.05). In NEG mode, 381 differential peaks were screened, of which 29 were MS2, 25 were upregulated, and 3 were downregulated (VIP>1, P<0.05). In POS mode, we screened 2457 differential peaks, of which 99 were MS2, 38 were upregulated, and 60 were downregulated (VIP>1, P<0.05).

### Individualization of Metabolomics Differences in COVID-19 Patients

**Figure 4:**
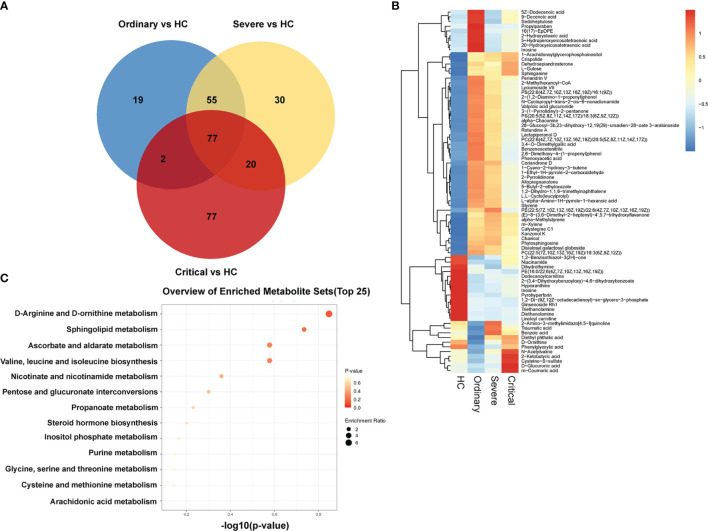
Massive differences of metabolism in sera of COVID-19 patients. **(A)** Venn diagram of negative and positive ion differential metabolites in the ordinary group, severe group, and critical group compared with healthy control. The number of differential metabolites is shown in each section. **(B)** Heat plot of the hierarchical clustering of differential metabolites. **(C)** Scatter plot of 13 differential metabolites with the most significant enrichment. *p*-value is plotted in a color map.

In order to obtain the differences of metabolites among different groups, 199 serum samples were analyzed for small molecule metabolites, and then the metabolites were clustered to form a well-defined compact cluster. These representative metabolites are also directly expressed in the second graph by the Venn diagram and heat map in **Figures 4A, B**. The color scale next to the heat map is the range of the expressed biomass. The vertical axis indicates the metabolites of our four groups; each row is a clinical test indicator. The metabolic patterns of metabolites were determined by cluster analysis (**Figure 4C**). The results of metabolic levels are represented as a heat map. By clustering metabolites with the same or similar metabolic patterns into classes, they can be used to infer the biological functions of known or unknown metabolites.

### Comparison of Metabolomics Differences Between Rehabilitation and Death

**Figure 5:**
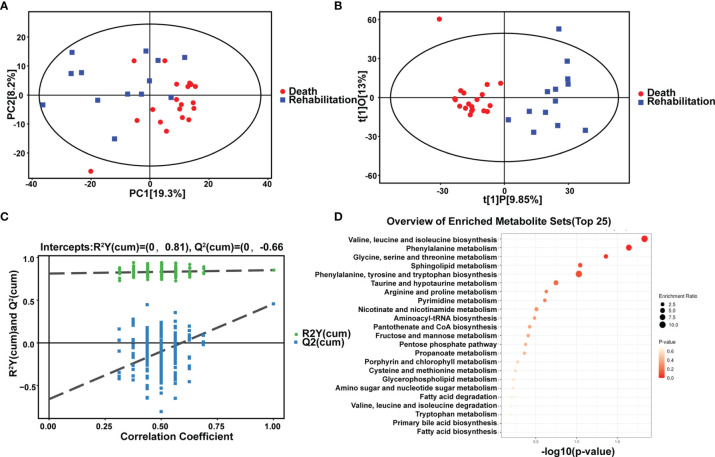
Comparison of metabolic differences between rehabilitation and death. Metabolomic alternations and comparison of metabolic differences between rehabilitation and death in the critical group. **(A)** PCA analysis. **(B)** OPLS-DA analysis. **(C)** Permutation analysis. **(D)** Scatter plot of 13 differential metabolites with the most significant enrichment. *p*-value is plotted in a color map.

For the first time, we compared differences in *in vivo* metabolism between critical rehabilitation patients and critical death patients. We conducted PCA analysis (**Figures 5A**–**C**) and hierarchical clustering analysis (**Figure 5D** and **Table 3**). We could see some differences from the PCA chart of the death group and the rehabilitation group (**Figures 5A**–**C**). There was a certain differentiation trend between the death group and the rehabilitation group, indicating that there was a certain difference in the metabolic levels of some samples between the two groups, and some samples had similar metabolic levels. OPLS-DA model analysis showed that the two groups were significantly different. Permutation tests indicated that the OPLS-DA model was not overfit, confirming that the differences between groups were partially significant (P<0.05). In NEG mode, we screened 679 differential peaks, of which 35 were MS2, 26 were upregulated and 9 were downregulated (VIP>1, P<0.05). Through hierarchical clustering analysis (**Figure 5D**), we considered that there was a metabolic difference between the two groups when p < 0.1. Therefore, it was found that in the recovery and death samples, their metabolic levels were mainly different in the pathways of the following small molecule metabolites: leucine, valine and isoleucine biosynthesis, phenylalanine metabolism, serine, glycine, and threonine metabolism, sphingolipid metabolism, and thenylalanine, tyrosine, and tryptophan biosynthesis. In POS mode, we screened 920 differential peaks, of which 66 were MS2, 21 were upregulated, and 45 were downregulated (VIP>1, P<0.05).

**Table 3:** Comparison of metabolic differences between rehabilitation and death.

Strategy	Metabolites (P<0.05)	Upregulated	Downregulated	Max (log2FC)	Min (log2FC)
Death VS rehabilitation	101	47	54	7.02	-3.12

### Association Analysis of Clinical Data With Metabolomics

There were many altered indicators in the patient’s blood test results (**Table S5**). The levels of many metabolites were altered in the patient’s serum samples, so it was tentatively determined that the metabolic pathways were not coordinated after SARS-CoV-2 infection. Inflammatory factors, such as IL-1 and IL-6, were significantly elevated in severe group patients. Notably, D-Dimer rose gradually and varied significantly from ordinary group patients to critical group patients (**Table S6**). In contrast, the increase in inflammatory response gradually decreased during hospitalization in parallel with clinical recovery in patients with mild COVID-19. Also, the altered number of cytotoxic T cells at the time of illness can be hypothesized to account for the major part of cellular immunity at the time of immunization, further leading to cytokine storms. Moreover, with the exacerbation of the disease, the non-specific immune response was found to be decreased (such as NK cells and so on) and the specific immune response was further weakened, implying that COVID-19 disrupts the body’s immune system.

**Figure 6:**
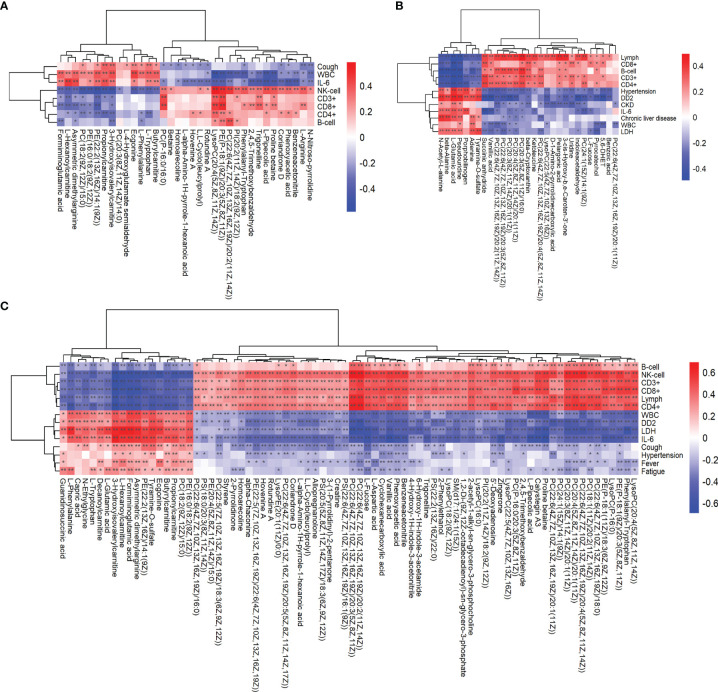
Association analysis of clinical data with metabolomics analysis. Heat plot of the hierarchical clustering of differential metabolites between **(A)** the critical group and severe group, **(B)** severe group and ordinary group, and **(C)** critical group and ordinary group. *p*-value is plotted in a color map. Data were analyzed by unpaired two-sided Welch's t test. *p < 0.05; **p < 0.01.

Combining clinical data, we analyzed the correlation between key metabolites and clinical information and their changing trends. Correlation analysis was performed between the differentially significant clinical data obtained through analysis and the differentially significant metabolite data obtained from the metabolomics analysis, where the redder the color indicated a stronger positive correlation between clinical results and metabolites, and the bluer the color indicated a stronger negative correlation between clinical results and metabolites. And we analyzed them in combination with the clinical blood test results on the vertical axis according to the color. For example, formiminoglutamic acid, N-acetyl-L-alanine, and guanidinosuccinic acid were associated with the expression of cytokines in the blood of COVID-19 patients (**Figures 6A**–**C**). These differential metabolites can be used as biomarkers to calibrate the disease process in COVID-19.

### Validation of the Biomarkers of Different COVID-19 Outcomes

**Figure 7:**
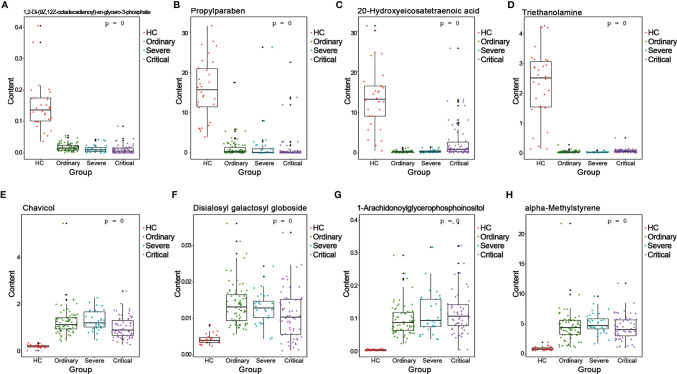
Validation of the biomarkers of different COVID-19 outcomes. ROC>0.7 of metabolites is selected as the biomarkers. **(A)** 1,2-di-(9Z,12Z-octadecadienoyl)-sn-glycero-3-phosphate. **(B)** 1-arachidonoylglycerophosphoinositol. **(C)** 20-hydroxyeicosatetraenoic acid. **(D)** Alpha-methylstyrene. **(E)** Chavicol. **(F)** Disialosyl galactosyl globoside. **(G)** Ginsenoside Rh1. **(H)** Propylparaben.

We analyzed the AUC values by ROC on the basis of positive and negative ion differentials in all comparison results. Differential metabolites with AUC values greater than 0.7 could be regarded as candidate biomarkers, and the results of ROC analysis for all differential metabolites are shown in **Tables S7**, **8**. The ROC analysis helped us to select a series of important biomarkers based on the ROC analysis, such as 1,2-Di-(9Z,12Z-octadecadienoyl)-sn-glycero-3-phosphate (**Figure 7A**), propylparaben (**Figure 7B**), 20-hydroxyeicosatetraenoic acid (**Figure 7C**), and triethanolamine (**Figure 7D**). They all had higher specific sensitivity in the HC group of sera. Furthermore, chavicol (**Figure 7E**), disialosyl galactosyl globoside (**Figure 7F**), 1-arachidonoylglycerophosphoinositol (**Figure 7G**), and alpha-methylstyrene (**Figure 7H**) showed higher specific sensitivity in the sera of COVID-19 patients. This is the first time experimental results have shown that these biomarkers could indicate different processes of COVID-19 outcomes.

## Discussion

The COVID-19 spread around the world is not optimistic. Research on epidemiological and clinical characteristics continues to accumulate our knowledge of COVID-19 (Prompetchara et al., 2020; Yi et al., 2020). Most patients have fever, cough, diarrhea, and other symptoms, but some patients also have acute respiratory failure, acute respiratory distress, septic shock, and other serious complications. Elevated inflammatory factors are considered as clinical early warning indicators for the treatment of severe and critical patients (Abebe et al., 2020; Abohamr et al., 2020; Zaim et al., 2020). One of the principal contributors to death in COVID-19 patients is cytokine storm. The occurrence of cytokine storm marks the start of a deregulated and malfunctioning immune response, which involves the sustained employment and expansion of lymphocytes and macrophages that secrete large amounts of cytokines, which may lead to a systemic inflammatory response, multiple organ failure, methemoglobinemia, and acute respiratory distress syndrome (Rabaan et al., 2021a; Rabaan et al., 2021b; Vanderbeke et al., 2021). However, a deeper understanding of the causes of cytokine storm in COVID-19 patients allows us to block its occurrence. We found that multiple APPs associated with cytokine control were significantly elevated in the sera of patients that had more aggressive outcomes, such as CRP, ORM1, and SERPINA3. On the other hand, some of the negative modulators of the inflammatory disorder resulted in the opposite outcome. Therefore, we hypothesized that the level of metabolism in COVID-19 patients was closely correlated with cytokine storm. Our results therefore displayed the metabolic characteristics of COVID-19 patients at different stages and revealed that ordinary, severe, and critical patients differed significantly from those of healthy individuals in their metabolism. For the first time, there were also remarkable variations in metabolism between recovered critical patients and dead critical patients. In the positive and negative ion modes, comparing the patients in the ordinary, severe, or critical groups, we found that the common metabolic differences were mostly concentrated in glycerophospholipids, fatty acids, and terpenoids.

Our metabolomic data showed that levels of many metabolites were altered in patient serum samples. Consistent with recent studies, we found that some metabolites such as glycerophospholipids, fatty acids, and terpenoids had significant changes in COVID-19 patients. In our research, we found that several glycerophospholipids were significantly reduced in the ordinary, severe, and critical groups of patients. This probably indicates that after SARS-CoV-2 infection, the energy produced by the patient’s metabolism decreases as the disease progresses, leading to a gradual decrease in the level of such metabolites. Importantly, it was shown that the oxidation of succinate played an important role in the replication of SARS-CoV-2 virus. Our results also revealed an increase in succinate with the course of the disease, suggesting that succinate is involved in regulating inflammatory signals that promote cytokine production throughout the progression of the disease. In addition, significant reductions in a range of amino acids were also found in our results, including glutamine, urea, citrulline, kynurenine, and others, which is similar to recent studies. The research also reported that the degree of increase in canine urinary quinoline indicates the level of IDO1 enzyme activation. Although our results demonstrated that COVID-19 patients and healthy controls showed significant differences at the metabolomic level, our current data were not able to confirm whether there were similarities between the metabolic disorders that may result from bacterial pneumonia, or other types of coronavirus infection, and those caused by COVID-19.

Our research also analyzed cytokines in COVID-19 patients, providing further evidence of the cytokine storm caused by SARS-CoV-2 infection. Our clinical data found that CRS-associated cytokines, such as a range of interleukins, IL-6, IL-1β, IL-10, and others, as well as interferons, increased dramatically with disease progression. On the contrary, for ordinary COVID-19 patients, an increase in the inflammatory response during hospitalization marked clinical recovery. Thus, the regulation of these cytokines may provide treatment for COVID-19 patient with novel ideas. There is growing evidence that the metabolic regulation of the body plays a very critical role in the release of cytokines. It has been found that the process of choline uptake and eventual metabolism of choline by the body is accompanied by the production of IL-1β and IL-18 in macrophages. However, increased uptake of α-ketoglutarate induces the release of IL-10, which in turn could suppress various chronic inflammatory conditions in the body and prolong life span. Glucose levels in the blood of patients with COVID-19 are significantly elevated, have a facilitative effect on SARS-CoV-2 virus replication, and induce cytokine production. In male COVID-19 patients, high canine uric acid is associated with the body’s immune response and is positively correlated with patient recovery. All these findings demonstrated that regulating the levels of various metabolites in patients could indirectly regulate the release of cytokines and had a greater impact on the treatment of COVID-19.

With the global COVID-19 pandemic and the trend of further challenges to world public health, it is particularly important that we are able to predict and detect the development of the disease and identify biomarkers. Many of the metabolite alterations found in our results corresponded to the severity of COVID-19. For example, it was shown that several inflammatory factors regulated by cytokines, such as CRP, ORM1, and SERPINA3, as well as IL-1b, TNF-α, and their associated cytokines, showed significant elevations in the critical group of patients. It was previously demonstrated that both S100A9 and S100A8 can induce the migration of inflammatory factors and immune cells in cells. In addition, CFI, one of the important components of the complement system, can act as a regulator of macrophage and T cell activation with LCP1/LPL. The negative regulators of some inflammatory factors, too, gradually decline with the degree of disease progression. This is similar to the results for our differential metabolites. Proteins such as CETP and APPs have also been previously studied and may be related with platelet function, including activation of the coagulation cascade. In the present study, combined with metabolomic data from patients with different disease courses of COVID-19 and their clinical baseline characteristics, we also hypothesized that the biomarkers we identified were involved in different immune responses and platelet and coagulation processes. Thus, our results are in agreement with previous clinical outcomes.

Amino acids are important substances for maintaining the normal function of the human body, and their metabolic processes are extremely important (Reeds, 2000). For example, glutamine is one of the most abundant amino acids in human plasma and cerebrospinal fluid. It is essential for the metabolism of various carbohydrates, proteins, and electrolytes, and is a class of substances necessary to stabilize the body to maintain normal functions (Psychogios et al., 2011). Therefore, alterations in amino acid metabolism in COVID-19 patients may lead to many clinical changes, and amino acid metabolites are also a potential biomarker. Of all the differential metabolites, the metabolism of tyrosine also deserves our focus. Tyrosine, a non-essential amino acid, is catabolized through various pathways by hydroxylation reactions with food and phenylalanine. A portion of tyrosine will be metabolized to dopamine, while another portion will be metabolized to dopamine, noradrenaline, and epinephrine (Grohmann et al., 2017). The majority of this tyrosine is metabolized to uronic acid, which is then catabolized to acetoacetate and cationic acid. A derangement in urine with high levels of tyrosine is diagnosed as tyrosinemia, which could eventually lead to severe liver dysfunction (Medina et al., 2014). In severe cases, it could lead to death and the disease is more prevalent in the pediatric population. Tyrosine is obtained through the hydroxylation of phenylalanine, or the hydrolysis of food proteins, which could be utilized in the biosynthesis of proteins, biogenic amines, and melanin. It is catabolized for energy through an enzymatic reaction of the species, producing acetoacetate and fumarate. The pathway of phenylalanine and tyrosine degradation is frequently described as a catabolic pathway that introduces aromatic amino acids to intermediates of the citric acid cycle. Adequate tyrosine is required for humans to metabolize other substances, which in turn produce key brain chemicals. When the metabolism is abnormal, symptoms such as vomiting, hepatosplenomegaly, oedema, ascites, and shortness of breath can occur, which are highly similar to those seen clinically in COVID-19 patients. The decomposition product of tyrosine, 4-hydroxyphenylpyruvate, a homologue of p-hydroxyphenylpyruvate, has been reduced in large amounts in COVID-19 patients, and the analysis might be associated to respiratory as well as hepatic metabolic disturbances.

Hypoxic airways are important and common divers of deterioration in patients with COVID-19. COVID-19 coagulopathy might be caused by thrombo-inflammation, and both microvascular thrombosis and damage to the pulmonary endothelium might lead to compromised airways and ultimately to a trend toward critical illness. Clinical studies have demonstrated that elevated levels of D-Dimer in the blood are a hallmark feature of coagulopathies. Therefore, elevated serum D-Dimer levels usually mark hypoxemia in patients and correlate with poor prognosis in COVID-19. And for some patients with coagulation disorders, such as diabetes, heart disease, hypertension, or some chronic kidney diseases, SARS-CoV-2 virus would be more likely to infect them. Our clinical data displayed that serum levels of D-Dimer were significantly elevated in COVID-19 patients as the disease progresses from ordinary, severe, and then critical, which was highly consistent with the current findings, and that the elevation of D-Dimer was due to plasma-associated hyperfibrinolysis. Hence the level of D-Dimer could predict the severity of the disease, as well as the mortality of the patient. Coagulopathy was a major common complication in COVID-19 patients, and the development of coagulopathy was due to elevated D-Dimer levels. In a study containing 560 cases, researchers found that in 260 of those cases, D-Dimer levels were abnormally elevated, representing 46.4% of the cases. Approximately 40% of patients with coagulopathy were ordinary COVID-19 patients, while nearly 60% were severe or critical patients. Zhang et al. also found in their study of 343 cases that the mortality rate of patients would be significantly reduced when the D-Dimer level exceeded 2.0 mg/L. In our study, we compared the levels of D-Dimer in critical rehabilitation and critical death patients, and the results were similar to the existing studies. In critical rehabilitation patients, with the clearance of SARS-CoV-2 virus and COVID-19 recovery, the level of D-Dimer gradually decreased. In contrast, in critical death patients, the levels of D-Dimer gradually increased with the progression of COVID-19 disease.

In summary, the sera metabolomics of COVID-19-infected patients with different courses reveals the pathogenesis of different courses after SARS-CoV-2 infection. And our research is the first to report the changes in metabolomics compared between patients who recovered and those who died with critical illness. In the correlation analysis of clinical symptoms with sera metabolism, we found that D-Dimer, liver function, IL-6, and other indicators were highly correlated with the degree of disease after SARS-CoV-2 infection. Furthermore, it is the first research where eight metabolites such as 1,2-di-(9Z,12Z-octadecadienoyl)-sn-glycero-3-phosphate, propylparaben, 20-hydroxyeicosatetraenoic acid, triethanolamine, chavicol, disialosyl galactosyl globoside, 1-arachidonoylglycerophosphoinositol, and alpha-methylstyrene were reported to be differentially expressed in patients with different courses of SARS-CoV-2 infection, suggesting that these differential metabolites could be used as biomarkers to calibrate the course of disease in COVID-19 patients.

## Data Availability Statement

The datasets presented in this study can be found in online repositories. The names of the repository/repositories and accession number(s) can be found in the article/**Supplementary Material**.

## Ethics Statement

The studies involving human participants were reviewed and approved by the Medical Ethics Committees, Zhongnan Hospital of Wuhan University. The patients/participants provided their written informed consent to participate in this study.

## Author Contributions

MG and CS designed the experiments. MG, YY, and CS wrote the manuscript. HP, YY, XZ, and FF collected the clinical samples and clinical data. LC, LH, XW, and YX performed the experiments and the statistical analyses. LC and LH read and revised the manuscript. MG and HP are the first co-authors and contributed equally to this article. All works were undertaken under the guidance of CS. All authors contributed to the article and approved the submitted version.

## Funding

CS acknowledges the financial support of the National Science and Technology Infrastructure Grants (NSTI-CR14-19). HP acknowledges the National Natural Science Foundation of China (81700493).

## Conflict of Interest

XZ was employed by the company Shanghai BIOTREE Biological Technology Co., Ltd, China.

The remaining authors declare that the research was conducted in the absence of any commercial or financial relationship that could be construed as a potential conflict of interest.

## Publisher’s Note

All claims expressed in this article are solely those of the authors and do not necessarily represent those of their affiliated organizations, or those of the publisher, the editors and the reviewers. Any product that may be evaluated in this article, or claim that may be made by its manufacturer, is not guaranteed or endorsed by the publisher.

## Supplementary Material

Supplementary Table 2 A total of 262 metabolites were identified from 5643 metabolite features extracted from the raw data acquired in negative-ionization modes by untargeted metabolomics analysis.

Supplementary Table 3 A total of 334 metabolites were identified from 7409 metabolite features extracted from the raw data acquired in positive-ionization modes.

Supplementary Table 4 A total of 77 common differentially expressed metabolites of negative ion differential metabolites in the ordinary group, severe group, and critical group compared with the healthy control group.

Supplementary Table 6 Baseline characteristics and clinical data of D-Dimer.

Supplementary Table 7 The negative mode results of ROC analysis for all differential metabolites.

Supplementary Table 8 The positive mode results of ROC analysis for all differential metabolites.
