# Supplementary material for: Sera Metabolomics Characterization of Patients at Different Stages in Wuhan Identifies Critical Biomarkers of COVID-19
Source: Front Cell Infect Microbiol. 2022 May 2;12:882661. doi: 10.3389/fcimb.2022.882661 (PMC9108257; doi:10.3389/fcimb.2022.882661)
Supplement: Supplementary file 1 [file Table_1.docx]

**Table S1. Demographics and baseline characteristics of COVID-19 patients.**

| Variables | Healthy Control  (N = 30) | COVID-19 | | |
| --- | --- | --- | --- | --- |
|  |  | Ordinary  (N = 59) | Severe  (N = 29) | Critical  (N = 28) |
| **Sex - no. (%)** |  |  |  |  |
| Female | 12 (40.0) | 30 (50.8) | 11 (37.9) | 10 (35.7) |
| Male | 18 (60.0) | 29 (49.2) | 18 (62.1) | 18 (64.3) |
| **Age - year** |  |  |  |  |
| Mean ± SD | 45.8 (12.2) | 53.2 ± 12.4 | 65.8 ± 18.1 | 62.3 ± 14.3 |
| Median (IQR) | 45.0 (38.2-53.2) | 56.0 (44.5-62.0) | 69.0 (57.0-81.0) | 62.0 (52.2-72.5) |
| Range | 23.0-68.0 | 21.0-77.0 | 21.0-89.0 | 31.0-87.0 |
| **SBP-mmHg** |  |  |  |  |
| Mean ± SD |  | 128.1 ± 15.5 | 131.0 ± 18.2 | 129.4 ± 22.6 |
| Median (IQR) |  | 125.5 (120.2-140.0) | 134.0 (116.0-148.0) | 130.0 (120.8-140.0) |
| Range |  | 99.0-179.0 | 99.0-160.0 | 65.0-180.0 |
| **DBP-mmHg** |  |  |  |  |
| Mean ± SD |  | 78.5 ± 12.4 | 73.0 ± 11.3 | 76.3 ± 12.5 |
| Median (IQR) |  | 78.0 (72.0-85.0) | 73.0 (68.5-79.2) | 80.0 (67.5-80.8) |
| Range |  | 18.0-110.0 | 50.0-100.0 | 40.0-100.0 |
| **Smoking history - no. (%)** |  | 1 (1.7) | 1 (3.4) | 2 (7.1) |
| **Time from Onset to Admission, Days** |  |  |  |  |
| Mean ± SD |  | 23.6 (11.0) | 11.8 (8.3) | 12.9 (7.3) |
| Median (IQR) |  | 28.0 (18.0-30.0) | 10.0 (5.0-15.0) | 10.0 (9.8-16.0) |
| Range |  | 1.0-48.0 | 1.0-35.0 | 3.0-30.0 |
| **Hospital stays** |  |  |  |  |
| Mean ± SD |  | 15.9 ± 8.6 | 33.5 ± 16.2 | 42.7 ± 15.7 |
| Median (IQR) |  | 13.0 (10.0-20.0) | 32.0 (21.0-43.0) | 39.0 (32.8-54.2) |
| Range |  | 4.0-46.0 | 9.0-82.0 | 18.0-68.0 |
| **Death** |  | 0 (0.0) | 1 (3.4) | 11 (39.3) |
| **ICU admission** |  | 0 (0.0) | 5 (17.2) | 27 (96.4) |
| **Symptoms - no. (%)** |  |  |  |  |
| Fever |  | 37 (62.7) | 23 (79.3) | 26 (92.9) |
| Cough |  | 35 (59.3) | 19 (65.5) | 26 (92.9) |
| Myalgia |  | 7 (11.9) | 9 (31.0) | 19 (67.9) |
| Fatigue |  | 19 (32.2) | 16 (55.2) | 20 (71.4) |
| Dyspnea |  | 13 (22.0) | 16 (55.2) | 24 (85.7) |
| Headache |  | 4 (6.8) | 2 (6.9) | 1 (3.6) |
| Abdominal pain |  | 1 (1.7) | 1 (3.4) | 2 (7.1) |
| Diarrhea |  | 4 (6.8) | 2 (6.9) | 4 (14.3) |
| Anorexia |  | 5 (8.5) | 2 (6.9) | 6 (21.4) |
| Haemoptysis |  | 0 (0.0) | 0 (0.0) | 1 (3.6) |
| **Comorbidity- no. (%)** |  |  |  |  |
| Hypertension |  | 10 (16.9) | 14 (48.3) | 11 (39.3) |
| Cardiovascular disease |  | 4 (6.8) | 4 (13.8) | 9 (32.1) |
| Cerebrovascular disease |  | 2 (3.4) | 4 (13.8) | 5 (17.9) |
| COPD/Asthma |  | 0 (0.0) | 4 (13.8) | 0 (0.0) |
| Chronic liver disease |  | 4 (6.8) | 6 (20.7) | 4 (14.3) |
| Malignancy |  | 2 (3.4) | 2 (6.9) | 3 (10.7) |
| Autoimmune disease |  | 2 (3.4) | 0 (0.0) | 2 (7.1) |
| Exposure seafood market |  | 0 (0.0) | 0 (0.0) | 1 (3.6) |
| **Complication** |  |  |  |  |
| Shock |  | 0 (0.0) | 0 (0.0) | 15 (53.6) |
| Acute cardiac injury |  | 1 (1.7) | 1 (3.4) | 17 (60.7) |
| Arrhythmia |  | 16 (27.6) | 10 (35.7) | 21 (75.0) |
| Sepsis |  | 1 (1.8) | 1 (3.8) | 23 (82.1) |
| ARDS |  | 0 (0.0) | 12 (41.4) | 26 (92.9) |
| Acute kidney injury |  | 2 (3.4) | 0 (0.0) | 12 (42.9) |
| Acute liver injury |  | 4 (6.8) | 5 (17.2) | 18 (64.3) |
| **Treatment - no. (%)** |  |  |  |  |
| Ordinary oxygen inhalation |  | 22 (37.3) | 26 (89.7) | 28 (100.0) |
| HFNO |  | 0 (0.0) | 2 (6.9) | 23 (82.1) |
| NIMV |  | 0 (0.0) | 0 (0.0) | 20 (71.4) |
| IMV |  | 0 (0.0) | 0 (0.0) | 16 (57.1) |
| ECMO |  | 0 (0.0) | 0 (0.0) | 5 (17.9) |
| CRRT |  | 0 (0.0) | 0 (0.0) | 11 (39.3) |
| Oseltamivir |  | 2 (3.4) | 5 (17.2) | 13 (46.4) |
| Arbidol hydrochloride |  | 42 (71.2) | 20 (69.0) | 17 (60.7) |
| Lopinavir/Ritonavir |  | 3 (5.1) | 7 (24.1) | 2 (7.1) |
| Glucocorticoid |  | 4 (6.8) | 16 (55.2) | 25 (89.3) |
| Antibiotics |  | 19 (32.2) | 23 (79.3) | 27 (96.4) |

**no. (%), number; SD, standard deviation; IQR, interquartile range; SBP, systolic blood pressure; DBP, diastolic blood pressure; ICU, intensive care unit; COPD, chronic obstructive pulmonary diseases; ARDS, acute respiratory distress syndrome; HFNO, High Flow Nasal Oxygen; NIMV, non-invasive mechanical ventilation; IMV, invasive mechanical ventilation; ECMO, extracorporeal membrane oxygenator; CRRT, continuous renal replacement therapy**
