# Supplementary material for: Sera Metabolomics Characterization of Patients at Different Stages in Wuhan Identifies Critical Biomarkers of COVID-19
Source: Front Cell Infect Microbiol. 2022 May 2;12:882661. doi: 10.3389/fcimb.2022.882661 (PMC9108257; doi:10.3389/fcimb.2022.882661)
Supplement: Supplementary file 5 [file Table_5.docx]

Table S5. Altered indicators in the patient's blood test results.

| Variables | Ordinary  (N = 59) | Severe  (N = 29) | Critical  (N = 28) |
| --- | --- | --- | --- |
| White blood cell, × 10^9^/L | 5.0 (4.3-5.8) | 6.3 (4.8-7.7) | 8.3 (6.0-11.7) |
| Red blood cell, × 10^12^/L | 4.0 (3.8-4.3) | 3.8 (3.3-4.3) | 3.9 (3.5-4.4) |
| Hemoglobin, g/L | 129.0 (120.1-138.7) | 113.8 (103.9-129.8) | 124.0 (108.2-137.5) |
| Neutrophil, × 10^9^/L | 2.8 (2.2-3.3) | 4.8 (2.8-6.5) | 7.4 (5.4-10.4) |
| Lymphocyte, × 10^9^/L | 1.4 (1.2-1.7) | 0.8 (0.5-1.2) | 0.6 (0.4-0.9) |
| Monocyte, × 10^9^/L | 0.5 (0.4-0.5) | 0.4 (0.3-0.7) | 0.3 (0.2-0.5) |
| Platelet, × 10^9^/L | 192.0 (169.2-220.5) | 219.0 (128.0-251.0) | 194.0 (150.2-243.8) |
| PT, s | 11.6 (11.1-12.2) | 12.5 (12.1-12.9) | 13.4 (12.4-14.1) |
| APTT, s | 32.3 (30.8-34.1) | 29.5 (28.1-32.3) | 30.0 (26.4-31.4) |
| D-Dimer, μg/mL | 125.5 (76.0-208.0) | 767.0 (241.0-5732.0) | 1149.5 (516.8-6031.8) |
| CK, U/L | 79.0 (58.0-101.0) | 52.0 (41.0-74.0) | 114.5 (45.5-256.5) |
| CK-MB, U/L | 10.0 (7.0-12.0) | 10.5 (7.2-13.8) | 19.0 (16.0-21.5) |
| LDH, U/L | 179.0 (154.0-197.0) | 200.0 (182.0-275.0) | 369.5 (271.0-592.2) |
| ALT, U/L | 26.0 (20.0-64.0) | 22.0 (12.0-47.0) | 42.0 (24.2-77.5) |
| AST, U/L | 24.0 (19.5-40.5) | 23.0 (20.0-38.0) | 52.0 (27.5-80.2) |
| TBIL, μg/mL | 12.8 (10.3-15.3) | 11.3 (7.7-13.9) | 15.0 (9.4-19.8) |
| Albumin, g/L | 39.9 (38.0-42.2) | 32.4 (29.2-39.4) | 28.9 (27.6-33.1) |
| BUN, mmol/L | 4.5 (3.8-5.4) | 6.5 (4.3-8.0) | 7.0 (5.2-8.9) |
| Creatinine, μg/mL | 57.5 (49.0-65.9) | 70.9 (57.1-88.4) | 67.6 (60.6-94.3) |
| CRP, mg/L | 1.9 (1.0-3.5) | 41.2 (17.6-70.2) | 68.4 (44.8-151.6) |
| PCT, ng/mL | 0.0 (0.0-0.0) | 0.0 (0.0-0.1) | 0.2 (0.0-0.8) |
| IL-6, pg/ml | 2.4 (0.4-4.9) | 7.6 (2.4-14.3) | 33.8 (13.6-87.3) |
| CD3^+^, cells/mL | 927.0 (796.0-1259.0) | 458.0 (335.0-710.0) | 328.5 (226.0-598.0) |
| CD4^+^ T cell, cells/mL | 598.0 (471.0-723.0) | 290.0 (183.0-416.0) | 182.5 (138.0-344.8) |
| CD8^+^ T cell, cells/mL | 346.0 (277.0-510.0) | 162.0 (83.0-377.0) | 132.0 (75.2-183.5) |
| B cell, cells/mL | 139.0 (103.0-234.0) | 91.0 (66.0-127.0) | 81.5 (42.0-153.8) |
| Nature killer cell, cells/mL | 217.0 (141.0-321.0) | 164.0 (91.0-209.0) | 41.5 (22.2-77.2) |
